# InstantDL: an easy-to-use deep learning pipeline for image segmentation and classification

**DOI:** 10.1186/s12859-021-04037-3

**Published:** 2021-03-02

**Authors:** Dominik Jens Elias Waibel, Sayedali Shetab Boushehri, Carsten Marr

**Affiliations:** 1 Institute of Computational Biology, Helmholtz Zentrum München - German Research Center for Environmental Health, Neuherberg, Germany; 2 School of Life Sciences, Technical University of Munich, Weihenstephan, Germany; 3 Roche Innovation Center Munich, Roche Diagnostics GmbH, Penzberg, Germany

## Abstract

**Background:**

Deep learning contributes to uncovering molecular and cellular processes with highly performant algorithms. Convolutional neural networks have become the state-of-the-art tool to provide accurate and fast image data processing. However, published algorithms mostly solve only one specific problem and they typically require a considerable coding effort and machine learning background for their application.

**Results:**

We have thus developed InstantDL, a deep learning pipeline for four common image processing tasks: semantic segmentation, instance segmentation, pixel-wise regression and classification. InstantDL enables researchers with a basic computational background to apply debugged and benchmarked state-of-the-art deep learning algorithms to their own data with minimal effort. To make the pipeline robust, we have automated and standardized workflows and extensively tested it in different scenarios. Moreover, it allows assessing the uncertainty of predictions. We have benchmarked InstantDL on seven publicly available datasets achieving competitive performance without any parameter tuning. For customization of the pipeline to specific tasks, all code is easily accessible and well documented.

**Conclusions:**

With InstantDL, we hope to empower biomedical researchers to conduct reproducible image processing with a convenient and easy-to-use pipeline.

## Background

Deep learning has revolutionised image processing [1]. For specific biomedical image analysis tasks such as cell segmentation [2, 3], cell classification [4–6] or in-silico staining [7, 8], deep learning algorithms now achieve higher accuracy than trained experts [6, 9, 10] and outperform humans at data processing speed and prediction consistency [11, 12]. However, machine learning algorithms are mostly developed to solve one specific problem. Moreover, applying them often requires a strong computer science and machine learning background.

We here provide InstantDL, a pipeline that automates pre- and post-processing for biomedical deep learning applications. InstantDL bundles commonly used deep learning algorithms in one easy-to-use framework. We did not change the bundled algorithms or tune them for a specific problem. Still, InstantDL achieves competitive results on different biomedical image datasets. It can be used for semantic segmentation (i.e. the classification of each pixel into a particular class), instance segmentation (i.e. the detection and classification of objects), pixel-wise regression (i.e. for in-silico staining) and image classification (i.e. to discriminate cancerous from healthy cells). InstantDL is an easy-to-use python package that can be executed by setting only two variables: the task and the data directory. For advanced users, twelve parameters can be set to adapt InstantDL to the specific task (see Methods). Moreover, different examples are provided in the documentation, which the user can follow. We provide 10 pre-trained models for transfer learning, since biomedical datasets are often sparsely annotated, as manual annotation is laborious and costly.

InstantDL is benchmarked on seven publicly available (see “Results” section) datasets: nuclei detection in divergent images [13], multi-organ nuclei segmentation [14, 15], lung segmentation from CT scans [16], in-silico prediction of a mitochondrial and a nuclear envelope staining [7], cell classification in digitized blood smears [17, 18], and cancer classification on histopathology slides [19]. Without any hyperparameter tuning, we achieve competitive results.

InstantDL serves as a centralized repository and provides a straight-forward wrapper to execute image computing tasks. It is designed for users interested in applying deep learning to their own data. A basic understanding of programming suffices. In contrast to easy-to-use browser based segmentation tools [3, 20], it is not limited to one specific task and ensures data privacy. The code is open source and well documented for those who want to customize the pipeline to their needs. By providing a debugged, tested, and benchmarked pipeline we help reduce errors during code development and adaptation, and contribute to reproducible application of deep learning methods.

## Implementation

InstantDL offers the four most common tasks in medical image processing: Semantic segmentation, instance segmentation, pixel-wise regression, and classification [21, 22]. In the following we describe the algorithms implemented in InstantDL to address these tasks and how they can be applied within the pipeline.

### Semantic segmentation

One of the standard approaches for detecting image patterns is semantic segmentation [23]. For each pixel in the input image a class label is predicted by the algorithm. The U-Net [24] is a commonly used architecture for semantic segmentation with numerous applications in biomedicine [25, 26]. It consists of a symmetric contractive path to capture context and an expansive path to capture fine localizations [24]. In InstantDL we made minor changes to the architecture from [24]: we (i) use padded convolutions to receive the same output and input dimensions and (ii) have implemented dropout layers in the encoder. These do not affect the performance of the network, but enable uncertainty quantification. The U-Net outputs continuous values between 0 and 1 for each pixel, which can be interpreted as probabilities to belong to a given class. InstantDL allows for two classes (background vs. foreground) and thresholds the output using Otsu’ method [27, 28].

### Instance segmentation

Instance segmentation is used to detect objects (instances) within an image [23, 28]. We implemented the Mask-RCNN [29] in InstantDL for this task. It first detects a bounding box for each object in the image and then performs a segmentation in each bounding box. Our Mask-RCNN is based on a ResNet50 from Abdullah's [30] implementation. The Mask-RCNN requires instance level ground truth: For each image in the training set, a set of labels has to be created, each containing a segmentation mask of one single instance. An algorithm to create instance level ground truth from binary semantic segmentation ground truth is provided as a jupyter-notebook with the pipeline.

### Pixel-wise regression

Tasks where no pixel-wise class labels but a continuous pixel value is desired (such as in-silico staining, [7]) are called pixel-wise regression. InstantDL uses the same U-Net implementation as for semantic segmentation. The only difference is that the U-Net output is not interpreted as probabilities to belong to one or another class, but is regarded as a regression. We thus use continuous labels for training and the mean-squared-error as regressive loss as proposed previously [7].

### Image classification

Here, the task is to classify each image into one of a specific number of given classes. For this task a residual network [31] is implemented. These architectures are widely used for biomedical image classification [12, 32]. Residual networks use residual blocks, a reformulation of layers as learning residual functions, which enable the use of many layers, while ensuring convergence [31]. We use a slightly modified ResNet50 with 50 layers in InstantDL, where we have added dropout layers to enable uncertainty estimation.

## The InstantDL pipeline

### Data preparation

**Fig. 1:**
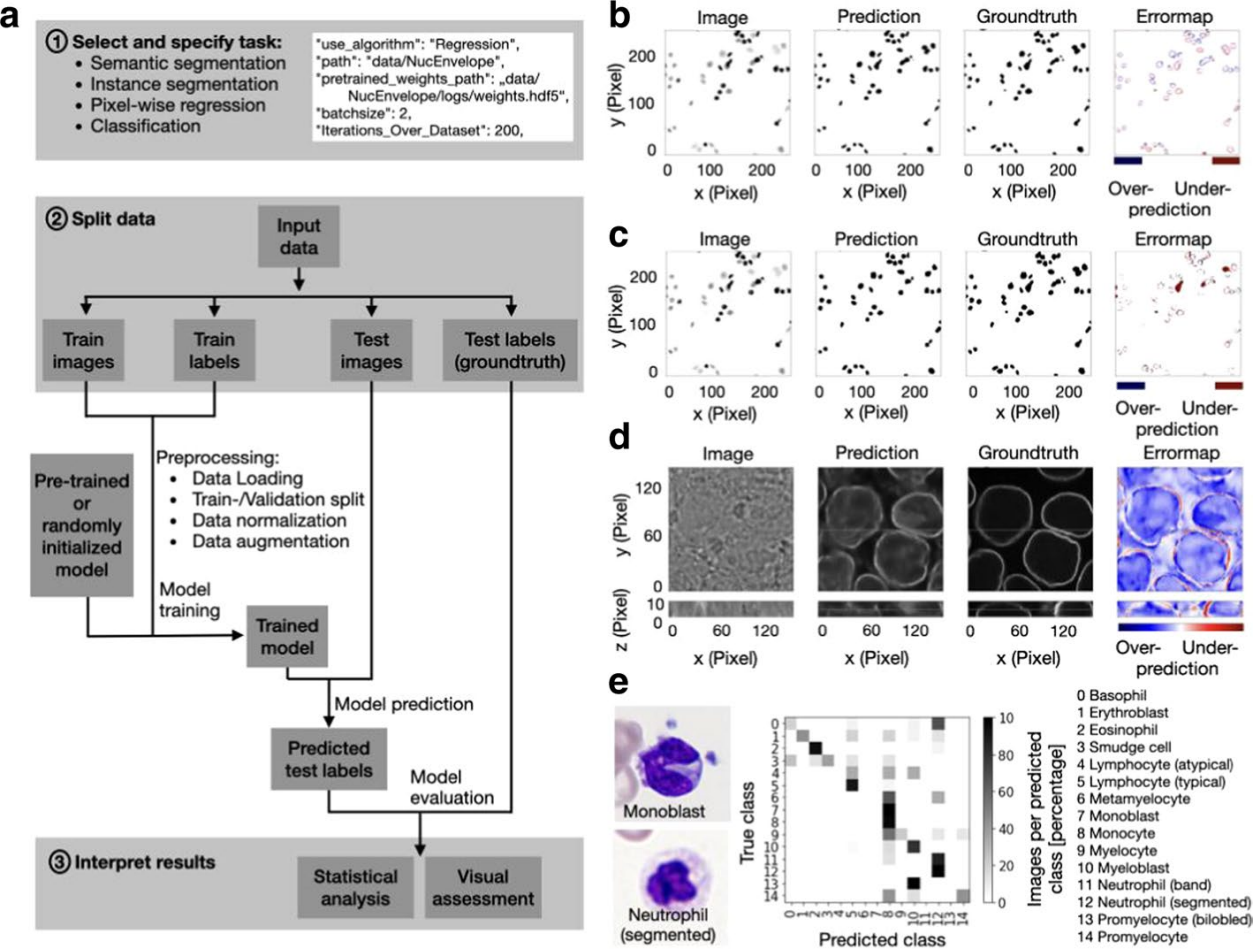
InstantDL provides an easy to use pipeline for the analysis of biomedical images. **a** Flow diagram of the pipeline with indicated user action highlighted in gray. (1) One out of four tasks (semantic segmentation, instance segmentation, pixel-wise regression, classification) is selected by the user. Up to twelve parameters can be set in the configuration file to adapt the pipeline to the task. A code snippet illustrates task selection and six of the twelve parameter settings in the configuration file: selected task (“use_algorithm”), path to folder (“path”), if pre-trained weights should be used the path to these (“pretrained_weights_path”) should be set, batch size (“batchsize”) and epochs chosen (“Iterations_Over_Dataset”). (2) Input data is split into train and test sets. The user specifies these by putting the data in the corresponding folders. After executing the python configuration file the pipeline will automatically load the data from the train folder, create a 20 percent validation split, normalize and augment the data (see Methods for details). Training is initiated with either a pre-trained or randomly initialized model. After training, the model predicts test labels: segmentation masks, pixel values or labels for the images in the test set according to the chosen task. (3) Results can be interpreted by the user via statistical and visual assessment of the predicted outcome by comparing it to the ground truth in the test set. **b** Example output for a 2D semantic segmentation task: Cell nuclei in a brightfield image (left) are segmented with InstantDL (Prediction) using the U-Net, and compared to the original annotation (Groundtruth). The Errormap indicates over- and under-predicted pixels. The image is part of the 2018 Kaggle nuclei segmentation challenge dataset [13]. **c** Example output for a 2D instance segmentation task (same image as in **b**): A binary mask is predicted for each object in the image using InstantDLs Mask-RCNN algorithm and compared to the groundtruth. **d** Example output for a 3D pixel-wise regression task using a U-Net. From stacks of bright-field images (Image) [7] the pipeline predicts a nuclear envelope (Prediction) that resembles the true staining (Groundtruth). The first row shows the x–y-plane, the bottom row the x–z plane of the 3D volume. **e** Example output for a classification task of benign and leukemic blood cells in blood smears from 200 individuals [17]. We show two exemplary microscopy images (left) of two white blood cell classes, a monoblast and a neutrophil. The white blood cell type is predicted with a ResNet50. The confusion matrix (middle) shows that most of the 15 classes can be well predicted, in accordance to Matek et al. [6]

Data has to be manually split in a train and a test set (see Fig. 1a) according to the user’s hypothesis: For one dataset a random split might be suitable [33], while for others a split on patient or tissue slide level is methodically appropriate [6]. InstantDL can process stacked images, enabling the prediction from multiple channels. Input and ground truth images must have the same filename including the file ending (Table 1).

**Table 1:** Overview on the image processing tasks implemented in InstantDL, required input, label, and output format

	Semantic segmentation	Instance segmentation	Pixel-wise regression	Classification
Input image	2D & 3D	2D	2D & 3D	2D
Labels	Binary images	Images with float pixel values	Images with float pixel values	Labels in a *.csv* file
Output	Binary images	Binary masks	Images with float pixel values	Labels in a *.csv* file
Architecture	U-Net	Mask RCNN	U-Net	ResNet50

### Pipeline settings

After data preparation the user can specify tasks and parameters in the configuration file (see Fig. 1a) or run InstantDL with default settings only providing the task and data path. A maximum of twelve parameters can be set. These are: The task (i.e. Semantic segmentation, instance segmentation, regression, or classification), the seed for reproducibility, the path to the project directory containing the train and test files, the pre-trained weights for model initialization, the batchsize, the number of iterations over the dataset (i.e. epochs), the data augmentations, the loss function, the number of classes, if the images should be resized during import, if the uncertainty should be calculated after training, and if the model should be automatically evaluated after training. Within the pipeline one.json file serves as a config file.

After setting these parameters, the user executes the configuration file which starts the training with InstantDL on the desired task using the training data with the pre-trained weights and the chosen configurations.

### Transfer learning

Pre-trained weights can be used to initialize a training process, a practice called transfer learning. The choice of weights can have a huge influence on the performance of the algorithm. Depending on the dataset and problem, transfer learning from natural images such as ImageNet to the medical domain can or cannot be the preferred solution [34]. With InstantDL we provide 10 pre-trained weight sets: four pre-trained weights from 2D nuclei segmentation (two from semantic and instance segmentation of the nuclear detection challenge dataset [13] and the nuclei in microscopy images of multiple organs segmentation, respectively [14, 15]), two pre-trained weights from 2D lung segmentation [16] (from semantic and instance segmentation), two from 3D in-silico staining [7] (from predicting mitochondria and the nuclear envelope from brightfield images), and one from the classification of white blood cells [17, 18] and metastatic cancer [19], respectively. Moreover, ImageNet weights can be loaded. InstantDL will load the weights given in the configuration file. It automatically selects weights for layers which fit the desired model and reports them in the logfile. As it is not clear which pre-trained weight will lead to a performance increase on the desired task we recommend to use weights from a task with similar characteristics to the dataset at hand in terms of structure and color. Expert users can choose to only train desired layers in the model file.

### Data augmentation

Data augmentation is a method commonly used in machine learning to artificially increase the variance in the training dataset and thereby train the network to generalize better [35]. We implemented spatial (horizontal and vertical flip, zoom and rotation) and color (contrast, brightness, poisson noise, feature scaling, standard-mean normalization, resampling, gamma shift) augmentations. The user can choose the desired augmentations, which are then randomly applied online, while importing the input images. The user can choose to visualize the augmentations used for training to ensure sensible inputs.

### Model training

InstantDL reads data from the corresponding folders and prepares for training and testing. This includes the following steps: (i) Model initialization with pre-trained weights, if selected. (ii) Import of data and normalization, split of validation data from the data contained in the train data folder, shuffle of training data, batch creation and online data augmentation. (iii) Training of the model using the Adam optimizer [36] for the given number of epochs using early stopping, which can be monitored live using tensorboard and automated saving of the best model. (iv) Prediction of labels from the test dataset. (v) For semantic segmentation, pixel-wise regression and classification uncertainty can be calculated after training.

### Model evaluation

The trained model is evaluated on the unseen test images and labels (i.e. the groundtruth). For semantic segmentation, instance segmentation and pixel-wise regression, the network predictions are saved as image stacks to ease evaluation of large datasets with limited CPU capabilities. This also allows an initial manual, qualitative evaluation and quality control. In the second step the predictions can be quantitatively evaluated. For that, accuracy, mean relative and absolute error, pixel-wise Pearson correlation coefficient and Jaccard index over all pixels of the test set are calculated. Boxplots are generated to visualize quantitative model performance. The standard quantitative evaluation output plots (i) the input images side-by-side to the corresponding labels and predictions and (ii) an error map between the labels and predictions to visualize training performance (see example evaluation Fig. 1b–d). For classification the predicted labels in the test set are compared to the true labels and multiple error scores (Jaccard index, mean absolute error, mean squared error, area under curve) are calculated. A confusion matrix and a receiver operating characteristic (ROC) curve are automatically visualized (Fig. 1e). All evaluation steps are implemented in the pipeline and can be set to be executed after testing. Additionally, post-processing (i.e. statistical analysis and visual assessment) is accessible in jupyter notebooks for customization, which are provided with InstantDL.

### Uncertainty quantification

**Fig. 2:**
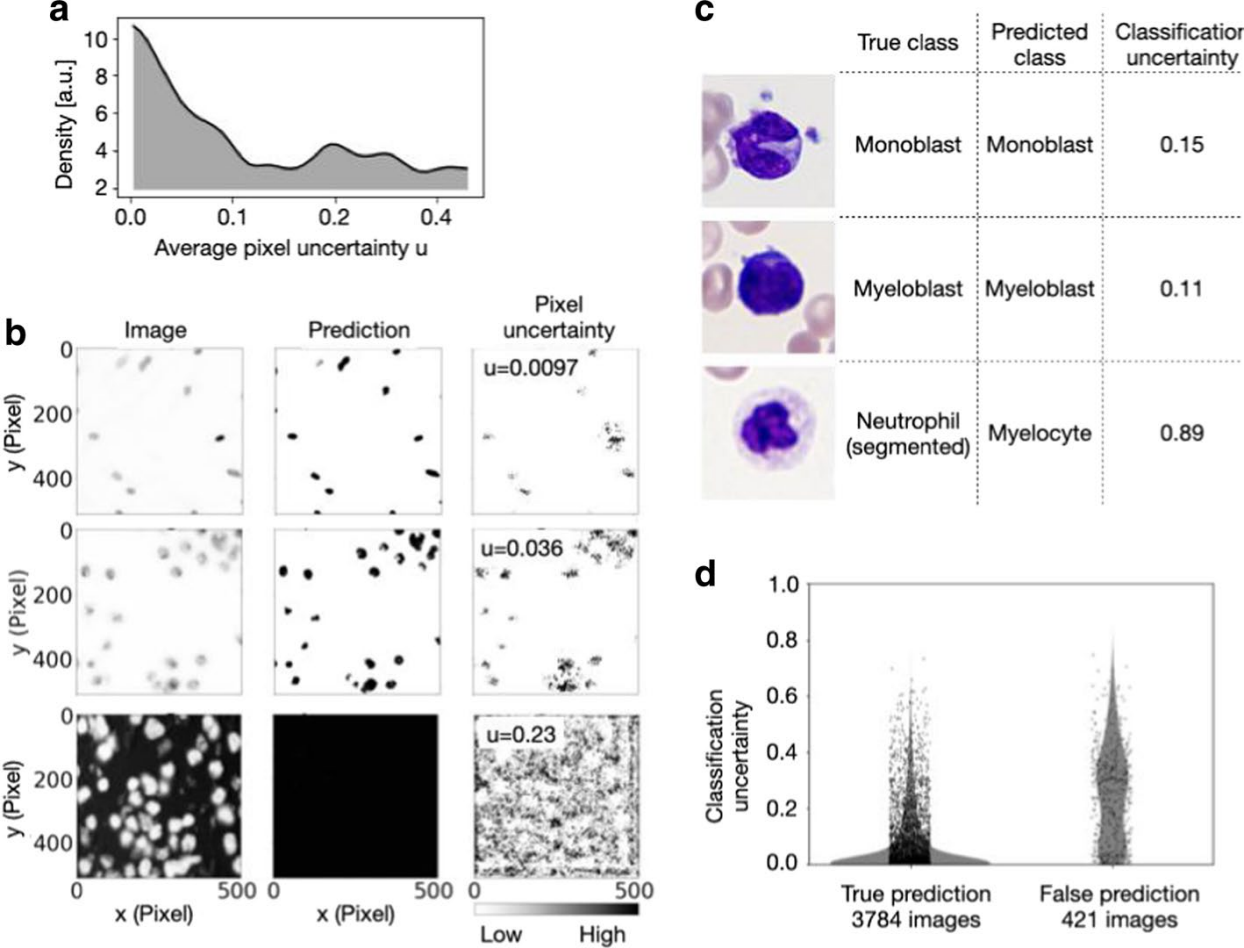
Uncertainty of semantic segmentation, pixel-wise regression and classification can be estimated with InstantDL. **a** The distribution of average pixel uncertainty u for 162 images of a semantic segmentation task [13]. The distribution is approximately bi-modal. **b** Three exemplary semantic segmentations from the data visualized in **a** with InstantDLs pixel uncertainty estimation. Correct predictions correspond to a low average pixel uncertainty u (top and middle row), while a high average pixel uncertainty indicates failed segmentations (bottom row). Regions with ambiguous predictions are indicated by high pixel uncertainty (right column). **c** For the prediction of white blood cells classes [6], classification uncertainty indicates incorrect predictions. **d** The distributions of classification uncertainty for correct and false predictions differ significantly (*p* value < 0.001, Mann–Whitney rank test)

Neural networks predictions can be unreliable when the input sample is outside of the training distribution, the image is corrupted or if the model fails. Uncertainty estimation can measure prediction robustness, adding a new level of insights and interpretability of results [37, 38]. Bayesian inference can be approximated in deep Gaussian processes by Monte Carlo dropout [37]. We have implemented Monte Carlo dropout for semantic segmentation, pixel-wise regression and classification in the pipeline. During the inference phase, 20 different models are created using Monte Carlo dropout and model uncertainty is calculated on the test set. For pixel-wise regression and semantic segmentation, the pipeline saves an uncertainty map. From this, pixel uncertainty can be plotted and saved to the project folder or average pixel uncertainty for an image can be calculated (Fig. 2a, b). For classification InstantDL adds the uncertainty score to the results file where a score close to zero indicates certain predictions, and score close to 1 indicates high uncertainty (Fig. 2c, d).

### Implementation of InstantDL

InstantDL is implemented in Python and leverages Tensorflow and Keras [39], which provide an excellent framework for our pipeline due to the modular, composable and user friendly design. The pipeline can be run locally ensuring data protection, on a cluster or with Google-Colab [40], which has successfully been used for deep learning projects [41] making it usable for those with limited computational resources. We provide the pipeline as one package in a Docker image [42] to simplify installation and application.

### Hardware requirements

InstantDL can run on a CPU with or without GPU acceleration, locally or on a server. For users with limited hardware InstantDL can also be run using Google Colab. The notebook to execute InstantDL in Google Colab is provided with the package. On an Nvidia GeForce RTX2070, the multi-organ segmentation dataset requires 8 min to train with semantic segmentation and 14 min with instance segmentation. Semantic segmentation with Google-Colab GPU took 9 min. This dataset contains 120 training images of size 512 × 512 pixels and we ran it with a batch size of 1 for 37 epochs. 

## Results

To evaluate InstantDL broadly, we applied it to seven publically available datasets (four of which come from data science challenges) and compared its performance to published results. If no test set was provided, we took 20% of the data to create our own test set. This was done on the highest level of abstraction, for example on patient level or tissue-slide level whenever possible, otherwise the data was randomly split. We used the same evaluation metrics as published in the respective papers (Jaccard index, AUC, Pearson correlation) to compare our results appropriately.

For pre-processing, we transformed the images to*.tiff* files and classification labels to a*.csv* file to adapt them to the InstantDL requirements. Training was performed by saving the best model using early stopping. As data augmentation we used horizontal and vertical flipping. For pixel-wise regression we used mean squared error loss, for semantic segmentation we used binary cross entropy loss and for classification we used categorical cross entropy loss. For instance segmentation, binary cross-entropy was used as segmentation loss in combination with the localization and classification loss in the Mask-RCNN [29].

**Fig. 3:**
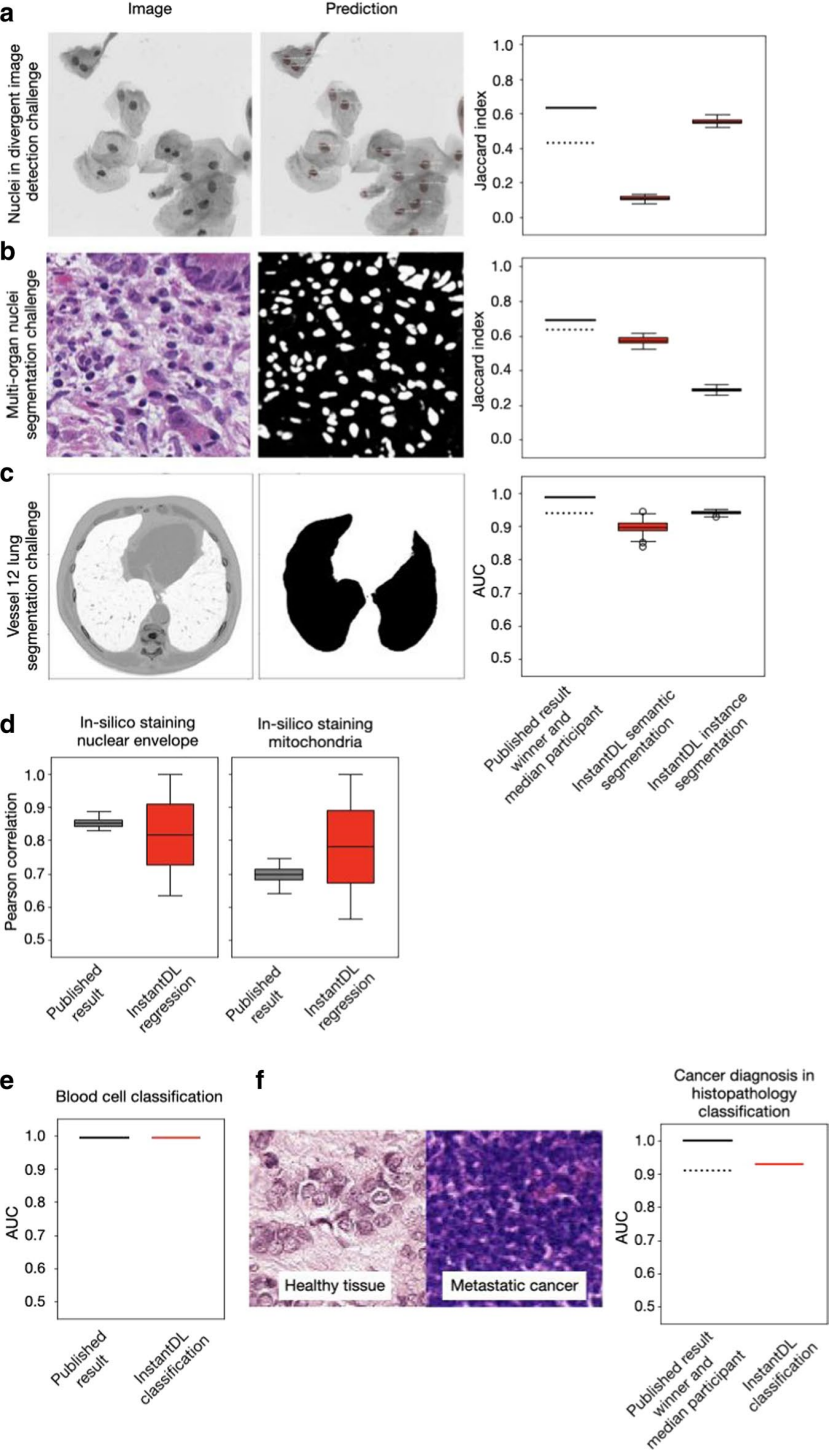
InstantDL achieves competitive performance on published datasets and computer vision challenges without hyperparameter tuning. **a** InstantDLs instance segmentation achieves competitive results on the nuclear detection challenge dataset [13], which contains a variety of experimental conditions, cell types, magnifications, and imaging modalities. We show one exemplary image from the dataset and the corresponding prediction using InstantDL’s instance segmentation. The winner of the challenge achieved a Jaccard index of 0.63 (solid line), while the median participant achieved 0.42 (dotted line). InstandDLs instance segmentation achieved a median Jaccard index of 0.60 without hyperparameter tuning. We estimate the Jaccard index distribution by bootstrapping, sampling 100 times half of the test set. Boxes indicate the median and the 25/75%ile of the distribution, whiskers indicate the 1.5 interquartile range. **b** For the challenge of segmenting nuclei in microscopy images of multiple organs with hematoxylin and eosin staining [14, 15], the winner achieved a Jaccard index of 0.69 (solid line) and the median participant 0.63 (dotted line). InstantDL using instance segmentation reached a Jaccard index of 0.29, and 0.57 using semantic segmentation. **c** Evaluation of instance segmentation of lung CT images from the Vessel-12 dataset [16]. The winner of the challenge reached an area under the receiver operating characteristic curve (AUC) of 0.99, while the median participant reached 0.94. InstantDL reached an AUC of 0.90 with semantic segmentation, and 0.94 with instance segmentation. **d** InstantDL’s pixel-wise regression performs similarly well as the published approach ([7] for in-silico staining of bright-field images in three dimensions, but with a higher variability. We achieved a median pearson correlation of 0.85 for nuclear envelope staining and 0.78 for mitochondria staining. **e** For classification of leukemic blast cell images vs. benign white blood cell images [17, 18], InstantDL achieved an AUC of 0.99, while Matek et al. report 0.99. **f** Classification of metastatic cancer in small image patches taken from larger digital pathology scans on histopathological images [19]. InstantDL achieved an AUC of 0.93 while the winner of the challenge achieved an AUC of 1.0 and the median participant 0.91

We evaluated the performance of semantic segmentation and instance segmentation on three datasets. In the first dataset we segmented nuclei in microscopy images contained in the Data Science Bowl 2018 [13] dataset. Using InstandDL instance segmentation we reached a median Jaccard index of 0.60 (25–75%ile: 0.61 to 0.58 estimated from bootstrapping), while using semantic segmentation we reached a median Jaccard index of 0.16 (25–75%ile: 0.15 to 0.17). The winner of the challenge reached a Jaccard index of 0.63 while the median participant reached 0.42 (solid and dotted line, Fig. 3a). The second task was the multi-organ nuclei segmentation challenge. Here, 30 microscopy images of various organs with hematoxylin and eosin staining are provided [14, 15]. We reached a median Jaccard score of 0.57 (25–75%ile: 0.56 to 0.59) with InstantDL’s semantic segmentation and 0.29 (25–75%ile: 0.28 to 0.30) with instance segmentation. The winner of the challenge reached 0.69 and the median participant scored 0.63 (solid and dotted line, Fig. 3b). Thirdly, we benchmarked InstantDL on lung CT images from the Vessel-12 challenge [16]. Using instance segmentation we reached an area under the receiver operator curve (AUC) of 0.94 (25–75%ile: 0.94 to 0.94), and 0.90 (25–75%ile: 0.88 to 0.92) with semantic segmentation. The winner of the challenge reached a score of 0.99 and the median participant 0.94 (solid and dotted line, Fig. 3c).

To evaluate InstantDL’s performance for pixel-wise regression, we predicted the 3D nuclear envelope and mitochondria staining from brightfield images [7]. For the nuclear envelope staining prediction, we achieved a median pixel-wise Pearson correlation to the real staining of 0.85 and 0.78 for the prediction of mitochondria staining (Fig. 3d), similar to the published result.

Classification performance was evaluated on two datasets. In Matek et al. images of single white blood cells from 100 leukemic and 100 non-leukemic individuals were classified into leukemic blast cell images vs. benign white blood cell subtype images [6]. We reached an AUC of 0.99 where Matek et al. reached 0.99 (Fig. 3e). The second task was to classify metastatic cancer image patches of digital pathology scans, taken from the Histopathologic Cancer Detection Kaggle challenge [19]. We reached an AUC of 0.93, while the winner reached 1.00 (solid line in Fig. 3f) and the median participant scored 0.91 (dotted line, Fig. 3f).

## Discussion

We present InstantDL, a deep learning pipeline for semantic segmentation, instance segmentation, pixel-wise regression and classification of biomedical images. InstantDL simplifies the access to the advantages of deep learning algorithms for biomedical researchers with limited computer science background. The only requirement is a solid understanding of the data (and how appropriately split it into training and test set), as well as of the task and loss function that should be optimized during training the model (see e.g. [1, 43]). The pipeline is designed for maximum automation to make training and testing as convenient and as easy as possible. However, some parameter settings depend on the dataset properties and therefore cannot be automated. After setting a maximum of 11 parameters, the pipeline can be run without further user interactions. We included state-of-the-art analysis metrics that are accessible out of the box. Moreover, we included uncertainty prediction to provide an additional level of interpretability of predictions.

We tested the performance of InstantDL on a variety of publicly available datasets and achieved competitive results without any hyperparameter tuning. While we could not reach the winners’ performance (typically using elaborate problem specific algorithms and data pre- and post-processing) of the respective image computing challenges with our out-of-the-box approach, the output of InstantDL suffices for standard biomedical data analytics performed after the image processing step.

To improve performance on a specific dataset we recommend to select specialized data augmentations and a suitable loss function in the configuration file for higher performance. Expert users can also adapt InstantDL’s code to their needs. We plan to extend InstantDL by implementing self-supervised and semi-supervised learning methods to utilize unlalleded data optimally in the future [34]. To improve semantic segmentation specifically, we will implement specialized loss functions, such as Malis loss [46]

The networks currently implemented are suitable for the most frequently used image processing tasks. Due to InstantDLs modular implementation it is however easy for users with python knowledge to exchange deep learning algorithms for a taylormade solution, e.g. other classifications networks like the ResNext [44] or MobileNet [45].

Other deep learning frameworks such as OpenML [46], ImJoy [47] and ZeroCostDL4Mic [48], nucleAlzer [3] and yeastspotter [20] allow for the application of deep learning methods on user data (Table 2). However, they all require data upload to a cloud system. OpenML, e.g., offers an online ecosystem of datasets and machine learning models, but the dataset will be made publically available with upload. The web tools nucleAlzer [3] and yeastspotter [20] are extremely easy to use, but provide only cell and nuclei segmentation using drag-and-drop with pre-trained machine learning models. ImJoy [47] and ZeroCostDL4Mic [48] are more flexible frameworks. ImJoy is an online tool where users can select plugins for processing their data, which is easy and convenient, yet not locally executable. Users can create their own plugins requiring professional programming knowledge. ZeroCostDL4Mic requires similar knowledge about programming as InstantDL, but solely relies on Google Colab. This requires the upload of data to the users Google drive, which has a memory limit of 15 GB in the free version and a runtime limit of 12 h. This can impose a hurdle for users, in particular for large datasets, common e.g. in computational pathology applications. InstantDL is applicable to any 2D or 3D image segmentation task and the only tool offering uncertainty estimation. Our pipeline can easily be installed and run locally on a computer or server, ensuring data scalability, privacy and security. However, InstantDL can also be used on cloud solutions, such as Google Colab. Table 2 compares the features of InstantDL with other frameworks.

**Table 2:** Comparison of InstantDL to other deep learning frameworks

	InstantDL	Open ML	ImJoy	ZeroCostDL4Mic
Host	Local, on cluster, or Google-Colab	Web based	Web based platform	Web based (Google-Colab)
Data privacy	Yes (running locally)	No (shared with upload)	Limited (hosted in the cloud)	Limited (hosted in the cloud)
Target audience	Researchers and developers	Researchers and developers	Biomedical researchers	Biomedical researchers
Developed for	Biomedical images	All kinds of data	All kinds of data	Biomedical images
Customizability of Code	Open source	Open source	Open source	Open source
Cost	Free	Free	Free	Free

## Conclusions

InstantDLs code is publicly available and convenient to tune and extend. It is ideal for labs looking for a customizable tool to apply deep learning to their own data. We thus  hope to empower biomedical researchers to conduct reproducible image processing with a convenient and easy-to-use pipeline.

## Availability and requirements

- Project name: InstantDL.
- Project home page: https://github.com/marrlab/InstantDL
- Operating system(s): Platform independent.
- Programming language: Python.
- Other requirements: cudatoolkit: 10.1.243 # in case of GPU existence, cudnn: 7.6.5 # in case of GPU existence, h5py: 2.9.0, hdf5: 1.10.4, imageio: 2.6.1, keras: 2.2.4, matplotlib: 3.1.1, numpy: 1.16.4, python: 3.6.7, scikit-image: 0.15.0, scikit-learn: 0.21.3, scipy: 1.3.0, tensorboard: 1.14.0, tensorflow: 1.14.0, tensorflow-gpu: 1.14.0 # in case of GPU existence, pandas: 1.0.3
- License: MIT.
- Any restrictions to use by non-academics: None.
- For reproducing our results, all data used for illustration and benchmarking is available for download at https://hmgubox2.helmholtz-muenchen.de/index.php/s/YXRD4a7qHnCa9x5.

## Availability and implementation

InstantDL is available under the terms of MIT licence. It can be found on GitHub: https://github.com/marrlab/InstantDL

## Supplementary information

Weights and validation data are available under: https://hmgubox2.helmholtz-muenchen.de/index.php/s/YXRD4a7qHnCa9x5

## Data Availability

The datasets generated and/or analysed during the current study are available in the HGMU repository, https://hmgubox2.helmholtz-muenchen.de/index.php/s/YXRD4a7qHnCa9x5 and the GitHub repository, https://github.com/marrlab/InstantDL

## Abbreviations

ROC: Receiver operating characteristic
AUC: Area under curve
